# Circumferential Fusion Employing Transforaminal vs. Direct Lateral Lumbar Interbody Fusion—A Potential Impact on Implants Stability

**DOI:** 10.3389/fsurg.2022.827999

**Published:** 2022-05-16

**Authors:** Andrey Bokov, Svetlana Kalinina, Andrei Leontev, Sergey Mlyavykh

**Affiliations:** ^1^Department of Oncology and Neurosurgery, Privolzhsky Research Medical University, Nizhny Novgorod, Russia; ^2^Department of Traumatology, Orthopedics and Neurosurgery, Privolzhsky Research Medical University, Nizhny Novgorod, Russia

**Keywords:** direct lateral interbody fusion, transforaminal lumbar interbody fusion, degenerative diseases, lumbar spine, screw loosening, hounsfield units

## Abstract

**Background:**

Different fusion techniques were introduced in clinical practice in patients with lumbar degenerative disc disease, however, no evidence has been provided on the advantages of one technique over another.

**The Objective of This Study:**

Is to assess the potential impact of circumferential fusion employing transforaminal lumbar interbody fusion (TLIF) vs. direct lateral interbody fusion (DLIF) on pedicle screw stability.

**Materials and Methods:**

This is a single-center prospective evaluation of consecutive 138 patients with degenerative instability of lumbar spinal segments. Either conventional transforaminal lumbar interbody fusion (TLIF) with posterior fusion or direct lateral interbody fusion (DLIF) using cages of standard dimensions, were applied. The conventional open technique was used to supplement TLIF with pedicle screws while percutaneous screw placement was used in patients treated with DLIF. The duration of the follow-up accounted for 24 months. Signs of pedicle screws loosening (PSL) and bone union after fusion were assessed by the results of CT imaging. Fisher‘s exact test was used to assess the differences in the rate of CT loosening and revision surgery because of implant instability. Logistic regression was used to assess the association between potential factors and complication rate.

**Results:**

The rate of PSL detected by CT and relevant revision surgery in groups treated with TLIF and DLIF accounted for 25 (32.9%) vs. 2 (3.2%), respectively, for the former and 9 (12.0%) vs. 0 (0%) for the latter (*p* < 0.0001 and *p* = 0.0043) respectively. According to the results of logistic regression, a decrease in radiodensity values and a greater number of levels fused were associated with a rise in PSL rate. DLIF application in patients with radiodensity below 140 HU was associated with a considerable decrease in complication rate. Unipolar or bipolar pseudoarthrosis in patients operated on with TLIF was associated with a rise in PSL rate while patients treated with DLIF tolerate delayed interbody fusion formation. In patients treated with TLIF supplementary total or partial posterior fusion resulted in a decline in PSL rate.

**Conclusion:**

Even though the supplementary posterior fusion may considerably reduce the rate of PSL in patients treated with TLIF, the application of DLIF provide greater stability resulting in a substantial decline in PSL rate and relevant revision surgery.

## Introduction

Degenerative stenosis of the lumbar spine is a frequently encountered condition in the aging population. Patients with spinal stenosis and segmental instability require decompression of nerve roots and fusion with pedicle screw fixation, which is the most effective solution in those cases (1, 2).

Different techniques were worked out to provide a fusion of altered segments, including PLF (posterolateral fusion), PLIF (posterior lumbar interbody fusion), TLIF (transforaminal lumbar interbody fusion), DLIF (direct lateral interbody fusion), and ALIF (anterior lumbar interbody fusion), however, no evidence has been provided on the advantages and superior outcomes of one technique over another. Even though TLIF is frequently supplemented by PLF to achieve circumferential fusion, those techniques are frequently opposed in relevant studies (3). DLIF using a lateral minimally invasive approach is getting more popular as an effective option to achieve indirect decompression and restoration of sagittal alignment (4). On the other hand, the evidence that the application of DLIF provides better outcomes than direct decompression with TLIF is insufficient especially if short fusion is required, therefore, no clear guidelines exist on the rational application of those techniques (5–8). An additional source of confusion is that the majority of studies focused on comparative analysis of various fusion techniques and the results are based on numeric scores, which can be strongly biased by different reasons that are irrelevant to the applied surgery (9–11).

Altered bone quality has a high prevalence in the elderly adult population and is associated with the most frequently reported complication associated with spinal instrumentation—implant instability development (12, 13). Taking into account concerns associated with a considerable upward trend in the number of fusions performed annually, an optimal surgical strategy should be worked out to decrease the complication rate. For now, there is some evidence that the application of cages with greater surface provides better distribution of load consequently it is expected that patients who are at risk of pedicle screw loosening development (PSL) may benefit from an application of broad cages (14, 15).

The objective of this study is to assess the influence of fusion type on the rate of implant instability development and associated revision surgery.

## Materials and Methods

This study is a non-randomized single-center prospective evaluation of consecutive 138 patients with degenerative diseases of the lumbar spine and instability of spinal segments, including 33 (23.9%) men and 105 (76.1%) women. The average age of participants at the time of operation was 56 years (SD = 8.7763; range 29–79 years). Patients with axial pain and neurogenic claudication or radiculopathy associated with spinal stenosis were enrolled. Participants underwent spinal instrumentations employing pedicle screw fixation either with transforaminal interbody fusion (TLIF) supplemented with posterior fusion (PF) or direct lateral interbody fusion (DLIF) during the period from 2012 to 2018. The duration of follow-up accounted for 24 months. Radiographic criteria of PSL were used to assess outcomes. This study was reviewed and approved by the local institutional board committee, given that no additional risks were anticipated; all patients signed informed written consent.

### The Inclusion Criteria Were

- Presence of degenerative disease of the lumbar spine with unstable spinal segments confirmed by functional radiograms or presence of low-grade symptomatic unstable spondylolisthesis,
- Radiculopathy or neurogenic claudication caused by degenerative diseases of the lumbar spine,
- Axial and radicular pain syndromes with visual analog scale (VAS) over 4 (0–10) and Oswestry Disability Index (ODI) over 40% resistant to repeated conservative treatment during 3 months or neurogenic claudication.

### The Exclusion Criteria Were

- High-grade spondylolisthesis (grades 3 and 4),
- Degenerative deformities that required correction of sagittal and frontal balance,
- Tumor-related lesions of the lumbar spine,
- Patients hospitalized for revision surgery,
- Cases with screw malposition and redirection detected on postoperative CT images,
- Patients with different types of fusion applied on different levels (hybrid constructs),
- Cases operated on more than two levels,
- Spinal instrumentation involving lumbosacral segment,
- Patients with the presence of pars interarticularis defects detected on CT images.
- Patients with excessive posterior decompression employing bilateral facet joints removal and laminectomy.

Before surgery, all patients underwent functional X-ray imaging and CT examination. The criterion for spinal instability was the difference in anterior translation on flexion-extension images >3 mm (16). The CT scans were performed from the T12-L5 levels using a single CT scanner (Aquilion 32, Toshiba Corporation). The scans used a slice thickness of 0.5 mm, covering a scan area of 50 cm. The scan parameters included tube voltage 120 kV, tube current 300 mA, auto mAs range 180–400; 1.0 s/3.0 mm/0.5 × 32, helical-pitch 21.0. Integrated software was used for calculations of bone density (Vitrea Version 5.2.497.5523) incorporating a window width/window level ratio of 2,000/500. During CT examinations, measurements of a vertebral body cancellous bone radiodensity in HU were obtained at the standard level of L3 in the sagittal, axial, and coronal planes. CT examination results were assessed by two independent certified radiologists. Measurements in the axial plane were taken at the level of the middle of the pedicles while those in the sagittal and coronal planes were taken along the geometric center of the vertebral body. Trabecular bone samples were selected using the maximal achievable square without traversing into the cortical bone to calculate bone density in each plane. Out of those figures, an average radiodensity was calculated for each case.

**Figure 1:**
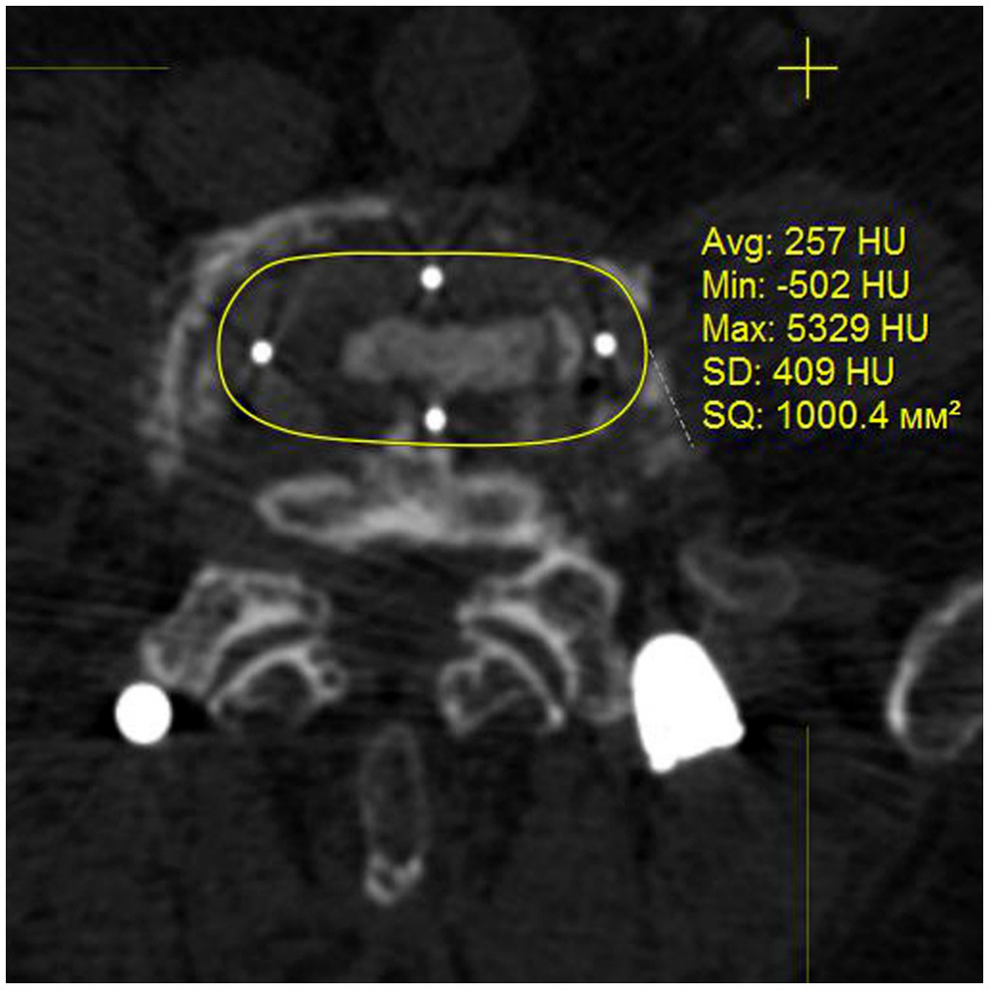
Cage used for DLIF, postoperative CT image in axial plane.

**Figure 2:**
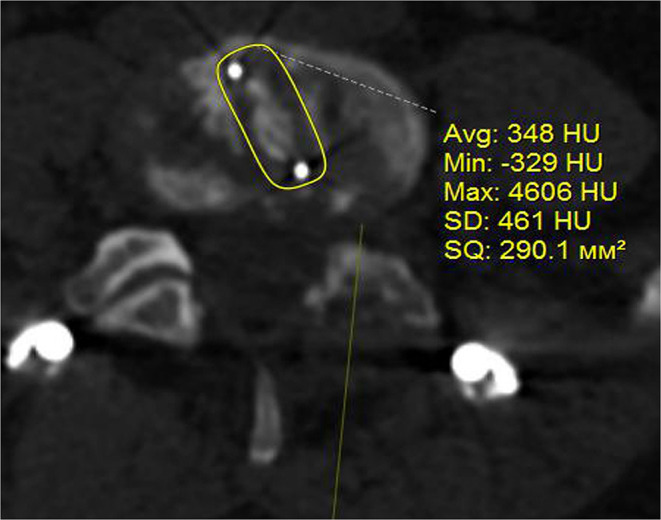
Cage used for TLIF, postoperative CT image in axial plane.

Either TLIF (75 cases−54.3%) with a single cage or DLIF (63 cases−45.7%) were used in this study. The allocation to DLIF or TLIF was based on the consensus of the committee of surgeons and the patient's consent (signed written consent was received from all patients). The applied technique of TLIF was a standard open one with unilateral facet joint removal, the applied DLIF technique was conventional as described previously (17). Cages of standard dimensions were used to perform DLIF and TLIF procedures with a footprint accounting for 1,000 mm^2^ for the former (Figure 1) and 290 mm^2^ for the latter (Figure 2). Autograft of locally harvested bone was used to perform TLIF while an allogeneic bone provided by the tissue laboratory of the institution was used for the DLIF procedure. Neither BMP nor other products that accelerate fusion formation were used in this study. The anterior longitudinal ligament has not been transacted during the DLIF procedure. Open TLIF was supplemented with posterior fusion in all cases while only in 15 (23.8%) cases treated with DLIF a posterior fusion was performed using tubular retractors. The technique of posterior fusion included the removal of facet joint capsules, cartilages, and decortication of the adjacent bone. Then the gap formed by capsule and cartilages resection was filled up with a locally harvested bone. Bilateral pedicle screw fixation with polyaxial screws was used in all cases, the applied technique was standard; a strait trajectory for screw placement was used. The conventional open technique was applied to supplement TLIF with pedicle screws while percutaneous screw placement was used in patients treated with DLIF. Pedicle screws were introduced at least to the anterior third of a vertebral body; bicortical screw placement was not used in the enrolled patients. The qualification of a surgeon was at least 7 years of experience.

The duration of the follow-up accounted for 24 months. All patients underwent clinical examination at the time of 3, 6, 12, and 24 months. CT examinations were performed at the time of 6, 12, months after surgery, and regardless of the time if clinical signs of implant failure signs were detected. CT examination was given at the time of 18 and 24 months if unipolar or bipolar non-union was detected according to the results of the former investigation. Interbody fusion was classified according to Tan classification as complete fusion, partial fusion, unipolar pseudoarthrosis, and bipolar pseudoarthrosis (18). Posterior fusion was assessed according to Christiansen‘s classification of fusion status as total facet joint ankylosing, partial ankylosing, and non-union (19, 20). Cases with PSL detected on CT images were registered. The criterion for screw loosening was a 1-mm or greater radiolucent zone around the screw, a double-halo sign, or both (21). Finally, patient outcomes were classified as either presence of PSL signs, regardless of the number of screws loosened, or the absence of this complication. Cases with PSL were subdivided into clinically significant and asymptomatic ones.

### Statistical Analysis

Two-tailed Fisher's exact test was used to test the statistical significance of the observed differences in the rate of PSL and revision surgeries applied. Students' *t*-test for independent samples was used to test the significance of the difference of means; *p* Values <0.05 were considered statistically significant. Shapiro-Wilk‘s test was used to test the normality of continuous data distribution. The association between PSL rate and potential risk factors was estimated using logistic regression analysis (a general multivariate logistic regression model was used). Statistica 12 (Statsoft) was used to perform calculations.

## Results

A total number of 138 patients with degenerative diseases of the lumbar spine were enrolled. The characteristics of the enrolled groups of patients are given in Table 1. According to the results of the analysis, no statistically significant differences were detected between the enrolled groups of patients.

**Table 1:** Characteristics of the enrolled groups.

**Characteristics**	**Group of patients treated employing DLIF, *n* = 63**	**Group of patients treated employing TLIF, *n* = 75**	**Statistical significance**
Age, years	M = 56	M = 58	*p* = 0.0705
	25–75% [74; 49]	25–75% [64; 51]	Mann-Whitney test
Male to female ratio	12:51	19:56	*p* = 0.4183 (two tailed Fisher's exact test)
Radiodensity, in Hounsfield Units	m = 125,1323 ± 5,0689	m = 118.2551 ± 4.2611	*p* = 0.2972
	SD = 40,2332	SD = 36.9020	(Student's *t*-test)
Number of patients with two level fusion	11 (17.5%)	18 (24.0%)	*p* = 0.4050 (two tailed Fisher‘s exact test)
Patients with radiodensity of cancellous bone below 110 HU	21 (33.3%)	35 (46.7%)	*p* = 0.1212 (two tailed Fisher‘s exact test)

By the end of the follow-up period, CT signs of PSL were detected in 27 (19.6%) patients, out of those only 9 (6.5%) were symptomatically deteriorating with axial pain VAS of more than 4 and ODI scores over 40; the latter 9 patients underwent revision surgery. Patients with clinically significant instability presented with either multiple pedicle screws instability or bilateral one-level screw loosening along with either unipolar or bipolar interbody pseudoarthrosis (Tan 3 or Tan 4) with complete posterior non-union. The primary analysis of the results with a breakdown by groups is given in Table 2.

**Table 2:** The initial analysis of the results.

	**Group of patients treated employing DLIF, *n* = 63**	**Group of patients treated employing TLIF, *n* = 75**	**Statistical significance**
PSL signs detected on CT images	2 (3.2%)	25 (33.3%)	*p* <0.0001 (two tailed Fisher‘s exact test)
Cases with symptomatic pedicle screws instability	0	9 (12%)	*p* = 0.0039 (two tailed Fisher‘s exact test)
Non-union after interbody fusion – Tan 3 and Tan 4	38 (60.3%)	36 (48.0%)	*p* = 0. 1722 (two tailed Fisher‘s exact test)
**Complete and partial posterior fusion**	**16 (25.4%)**	**41 (54.6%)**	***p*** **=** **0.0018 (two tailed Fisher‘s exact test)**

*Bold values were given to highlight statistically significant values*.

According to the results given in Table 2, the rate of pedicle screw loosening detected on CT and the rate of revision surgery was greater in the group of patients treated with TLIF. Relatively high prevalence of CT loosening signs, anterior and posterior non-union can be explained by a considerable proportion of patients with radiodensity below the threshold of 110 HU which corresponds to 90% specificity of osteoporosis detection. The number of those cases accounted for 56 (40.5%) in the total cohort of enrolled patients. It was expected that the application of autograft may favor interbody fusion formation in patients treated employing TLIF, however, the difference between two groups in Tan 3 and Tan 4 rate pseudoarthrosis turned out statistically insignificant. It should be mentioned, that in 16 cases a spontaneous posterior fusion was evaluated in the group of patients treated with DLIF.

To estimate a relative contribution of the applied fusion technique to screw loosening, to detect other contributing factors, and to assess their interaction, a general logistic regression analysis was used. The dependent variable was the presence of CT signs of PSL. Finally, the model with the best subsets of variables that provides the best explanatory value was chosen. Mining the data, it has been estimated that the best model can be estimated only if higher-order effects were taken into account. The parameters of the estimated general multivariate logistic regression model with the best characteristics are present in Table 3.

**Table 3:** Parameters of the estimated logistic regression function.

**Components of regression model**	**Regression coefficient and its statistical significance**	**OR per unit change with 95% CI**
Intercept	0.5594	
	*p* = 0.7701	
**Radiodensity in HU**	**0,0356**	**0.9650**
	***p*** **=** **0.0077**	**[0.9399; 0.9909]**
**Number of levels fused**	**1.9043**	**6.7148**
	***p*** **=** **0.0206**	**[1.3193; 31.1754]**
**DLIF application in patients with cancellous bone radiodensity below 140 HU**	**−3.7270**	**0.0241**
	***p*** **=** **0.0182**	**[0.0011; 0.5455]**
**Unipolar and bipolar pseudoarthrosis in patients treated with TLIF**	**2.5825**	**13.2308**
	***p*** **=** **0.0018**	**[2.6669; 65.6408]**
**Partial and total posterior fusion in patients treated with TLIF**	**−3.4008**	**0.0334**
	***p*** **=** **0.0010**	**[0.0045; 0.2445]**
Unipolar and bipolar pseudoarthrosis in patients treated with DLIF	0.5482	1.7302
	*p* = 0.6170	[0.1988; 15.0560]
Partial and total posterior fusion in patients treated with DLIF	−0.0299	0.9705
	*p* = 0.9820	[0.0708; 13.3068]

*Bold values were given to highlight statistically significant values*.

The overall goodness of fit of the estimated general multivariate model was χ^2^ = 69,722, *p* < 0.0001. According to the results of the analysis, a decrease in radiodensity values and a greater number of levels fused were associated with a rise in pedicle screw instability development rate. DLIF application in patients with radiodensity below 140 HU was associated with a considerable decrease in PSL rate. Unipolar or bipolar pseudoarthrosis in patients operated on applying TLIF was associated with a rise in the PSL rate while non-union grade 3 and 4 was not associated with an increment in PSL rate in a group of patients treated with DLIF. In patients treated with TLIF, a supplementary total or partial posterior fusion resulted in a decline in PSL rate conversely this factor turned out insignificant in patients treated with DLIF. The estimated logistic regression model had a specificity accounting for 95.5%, sensitivity of 68.0%, and preciseness of classification 90.4%.

## Discussion

Even though pedicle screw fixation with interbody fusion has been proven to be the most effective treatment option for patients with spinal stenosis and segment instability, the rate of instrumentation failure caused by altered bone quality remains considerable given the high prevalence of the latter in the elderly adult population (21–23). Different diagnostic tools are used to detect patients who are at risk of implant instability development and the application of radiodensity in HU becomes popular because those figures correlate with bone mechanical properties. Furthermore, it has been defined that thresholds of 110 HU and 135 HU have maximal specificity for osteoporosis and osteopenia detection, respectively (24–26). The initial analysis demonstrates a high prevalence of cases with altered bone quality that accounted for 54 (41.2%) in the enrolled group. Those figures explain a relatively high rate of screw loosening and non-union detected during the follow-up period.

To achieve substantial stability of the altered segment various types of interbody fusion were suggested, out of those the most frequently used are PLIF, TLIF, DLIF, OLIF, and ALIF (4). Despite a considerable number of relevant works published, no clear guidelines were worked out for the rational application of those techniques. The source of additional confusion to the reported results is that a hefty majority of relevant studies are based on the dynamics of subjective numeric scores assessment. Apparently, those studies have evident weak points. Firstly, the application of numeric scores is not standardized yet (8). Secondly, the results of those studies can be influenced by many irrelevant to the applied surgery causes, including the accuracy of diagnoses, socioeconomic, behavioral, psychological factors, sacroiliac joints dysfunction, and adjacent level degeneration (9, 10, 27, 28). To avoid bias relevant to the application of subjective numeric scores, radiographic signs of PSL were used in the current study. Considering that signs of CT loosening can be asymptomatic, questioning their clinical relevance, the rate of clinically significant loosening that requires revision surgery was taken as an additional criterion for the assessment of the results.

According to the results of research on biomechanics, the most reliable mechanism of PSL are micro-movements caused by craniocaudal toggling and rotational stress that increase the load to the zone of the bone-screw interface (29). To minimize micro-movements that cause PSL the application of the most stable type of fusion is required. By using biomechanical tests some evidence has been provided, that application of broad cages may lead to better load distribution, decreasing stress on screws, rods, and endplates (13, 30). The results of our study confirm the clinical relevance of biomechanical studies since a considerable decline in the rate of PSL detected was associated with DLIF application. The observed effect achieves maximum in patients with radiodensity of cancellous bone below 140 HU. According to our results, unipolar or bipolar pseudoarthrosis is a significant factor promoting PSL in patients operated on applying TLIF while those treated with DLIF tolerate delay in interbody fusion formation. Although posterior fusion is frequently opposed to interbody fusion, it has been defined that circumferential fusion using both listed provides a greater success rate in patients with degenerative diseases of the lumbar spine (31–34). Our findings demonstrate that total and even partial posterior fusion is associated with a decline in PSL rate if TLIF was employed, conversely, posterior fusion turned out to be an insignificant factor in patients treated with DLIF, consequently, additional posterior fusion is not required in this group of patients.

Eventually, the main findings of the analysis demonstrate that the application of DLIF may provide a considerable decline in the rate of PSL detected by CT, especially in patients with radiodensity below 140 HU. Those findings can be explained by a beneficial distribution of forces alleviating stress on the screw-bone interface. A statistically significant difference in clinically significant loosening rate supports the conclusion that the observed effect of DLIF application is clinically relevant and has thepotential as a beneficial option in patients who are at risk of implant instability development.

## Limitations

This study has limitations that should be acknowledged. Firstly, this study is not a randomized one; secondly, the number of participants is relatively small to provide a robust regression model suitable for instrumentation failure prediction. On the other hand, the results of the study provide evidence that the application of DLIF with a broad cage results in a decline in the rate of pedicle screw loosening and associated revision surgery. Also, a potential bias was addressed in this study associated with heterogeneity in bone properties, number of levels fused, and application of supplementary posterior fusion.

## Conclusion

Even though the supplementary posterior fusion may considerably reduce the rate of pedicle screw loosening in patients treated with TLIF, the application of DLIF provide greater stability resulting in a substantial decline in PSL rate and relevant revision surgery.

## Data Availability Statement

The raw data supporting the conclusions of this article will be made available by the authors, without undue reservation.

## Ethics Statement

The studies involving human participants were reviewed and approved by Local IRB Committee of Privolzhsky Research Medical University. The patients/participants provided their written informed consent to participate in this study.

## Author Contributions

AB contributed to study concept and design. SK contributed to data collection, data mining, and manuscript editing. AL contributed to data collection and data mining. SM supervised the project and reviewed the manuscript. All authors contributed to the article and approved the submitted version.

## Funding

This study was supported by the state assignment (Theme No. 121030100311-3).

## Conflict of Interest

The authors declare that the research was conducted in the absence of any commercial or financial relationships that could be construed as a potential conflict of interest.

## Publisher's Note

All claims expressed in this article are solely those of the authors and do not necessarily represent those of their affiliated organizations, or those of the publisher, the editors and the reviewers. Any product that may be evaluated in this article, or claim that may be made by its manufacturer, is not guaranteed or endorsed by the publisher.
